# Health of Soldiers

**Published:** 1861-09

**Authors:** 


**HEALTH OF SOLDIERS.**—A watch-fob, five-by-six, three-ounce flexible-covered volume was published in June, which is worth all the books yet issued for the army for its moral, physical, and religious interests. The editor wrote the book, but feels no hesitancy in giving it this high praise, because it was taken mainly from the Bible, Watts's Hymns, and the *Scientific American*—all good authority. In addition, it contained a complete system of cookery, of which the world-renowned Soyer was the author. The remainder of the little volume is made up of practical suggestions taken from standard medical writers. We claim nothing in it for ourself, as our own, except the wording of some things in such a way that the most uneducated person might understand them, without giving him the headache. Every soldier ought to have one of these books. Every reader of this JOURNAL, who has a friend or relative in the army, should order one for such friend or kinsman; the cost, post-paid, is twenty-five cents. And for the not a few who are alone in the world, homeless, friendless, fatherless, some man of means should purchase a few hundred or thousand at ten cents, for gratuitous distribution.

We feel perfectly assured that the same amount of money which has been expended in purchasing hymn-books, "soldiers' libraries," etc., would have done a hundred-fold more good to the army and the country in the gratuitous distribution of our book on "SOLDIER-HEALTH," because its first object was to keep the soldier well, and to teach him how to manage when left alone, or lost, wounded or sick. Nine tenths of the men in the army have had more or less of a religious education, and already understand in some small measure the fundamental principles of our holy religion; but as to the method of preserving their health, and thus keeping themselves in the very highest state of military efficiency, and the means of meeting the various casualties of war, when thrown upon their own resources, they are most profoundly ignorant; hence our book meets the first, fifth, and fiftieth want of the times.

Very recently, a poor soldier was found to have bled to death in the woods, from a wound in the thigh. He had crawled to a little shelter a few rods distant from the battle-field, yet the knowledge imparted in four lines of our book would have shown him how easily life might have been preserved with a little stick, or bayonet, or ramrod, and a string or piece of shirt or trowser. And there are fifty-eight other short items of instruction as to the best means of action in emergencies. The book earnestly advises that before a march, or battle, or other hard day's work, an early breakfast should be given to the men, and each canteen should be filled with cold coffee. The neglect of this on the part of the men—for three days' provision had been dealt out to each soldier the evening before—was a main cause of the panic at Bull Run, for the army began their march before daylight, each individual man having neglected to prepare his breakfast or coffee, and no opportunity for doing so occurring during the march, a battle was fought and really won on an empty stomach; but, when famishing for food, and the physical energies were exhausted by a twelve hours' march and fight on one of the hottest days of midsummer, the very best preparation possible was made for the excitement of an uncontrollable consternation, by the occurrence of any one of a million trivial but very possible incidents. Besides the hunger, there was the more urgent and tormenting thirst, which the flat water of a canteen could not satisfy, even if there had been a plenty of that; while a sip of strong coffee would not only have slaked the thirst in a measure, but would have imparted invigoration, and encouragement, and confidence to the men, for want of which the victory was lost.

As an argument for not touching alcohol in any form, at least not until the work was done, it was stated on page 37, that an important revolutionary battle was lost by the drunkenness of a single man, as reported in some histories. On the 29th of July, the morning papers stated, without contradiction since, that an officer of high grade was found so drunk at Bull

Run, that he was superseded on the field; and that having failed to do a certain work with the seven thousand men under him previous to the battle, the use of these men was lost; and had the work assigned been done, the panic would have been promptly arrested.

On the same day, documentary evidence was presented in Congress, that several millions of dollars' worth of property had been lost to the nation, by the apparent "drunkenness" of a navy officer.

The Press for many weeks has been teeming with accounts of the discomfort, and privation, and actual sufferings of the men, in consequence of the almost universal ignorance of cookery. Our book directed special attention to this most important subject, and embodied the experience of the best cook in the world, in the British Army, so that no man need be at a loss to cook for himself, for his mess, or for a hundred or thousand men, without making any material mistake.

AN EVIL TO COME, and which will come with the inevitability of the sun's shining, is provided against with the most perfect certainty in "SOLDIER HEALTH;" it is epidemic autumnal diseases, diarrhea and dysentery, which will begin to make their approach when hot days are followed by cool nights. These maladies have been known to frustrate campaigns and paralyze whole armies in a night, but which could have been avoided by encamping a little higher up the mountain, or on the other side of a stream.

An intelligent officer stated that he left his native city with a thousand and fifty men, and returned within a year with barely five hundred, from Mexico, the remainder having been left in hospitals, or died on the march, for they never came in sight of an enemy. These things show that the health of the soldiers is emphatically of the very first importance; hence a watch-fob volume, which can be taken into the battle-field without its being an appreciable inconvenience, containing, as "SOLDIER HEALTH" does, so much instruction, (moral, medical, and surgical,) applicable to such a situation, especially in the event of being wounded and left behind or for dead, when such is not the case, such a volume, we say, can be made, under a multitude of possible contingencies, worth more than its weight in gold to the suffering soldier. And now that a new army is collecting "for three years or during the war," the book becomes of multifold more importance, especially at this juncture when attention to one single recommendation would prevent an incalculable amount of disease and suffering and very many deaths; that is, the wearing around the abdomen (belly) a woolen flannel bandage about fourteen inches broad, and long enough to be double in front, as a means of supporting the strength, imparting warmth, and of averting cold on the bowels, thus keeping off diarrhea, dysentery, and cholera, which are the diseases of all armies, especially in the fall of the year. That this simple expedient will do it, some of the foreign regiments who have experienced its efficacy in their own persons, it being a standing regulation in Germany, especially in Prussia, have adopted it almost to a man, of their own accord.

Having given as many of our books to the army as we felt able to do, it is now offered by the hundred to those who wish to distribute it gratuitously in the army, for ten dollars, or ten cents each; two dollars a dozen, twenty-five cents singly. As a coïncidence, it may be mentioned that of the whole Bible the portion selected for the soldier's reading in times of peril or deliverance was that which Luther always sang when rocked in storms, to wit, the forty-sixth psalm.

